# A CRM1 Inhibitor Alleviates Cardiac Hypertrophy and Increases the Nuclear Distribution of NT-PGC-1α in NRVMs

**DOI:** 10.3389/fphar.2019.00465

**Published:** 2019-05-07

**Authors:** Zuheng Liu, Haiping Tian, Jinghai Hua, Wanqiang Cai, Yujia Bai, Qiong Zhan, Wenyan Lai, Qingchun Zeng, Hao Ren, Dingli Xu

**Affiliations:** ^1^State Key Laboratory of Organ Failure Research, Department of Cardiology, Nanfang Hospital, Southern Medical University, Guangzhou, China; ^2^Key Laboratory for Organ Failure Research, Ministry of Education of the People’s Republic of China, Guangzhou, China; ^3^Department of Cardiology, The Affiliated Hospital, Inner Mongolia Medical University, Hohhot, China; ^4^Department of Rheumatology, Nanfang Hospital, Southern Medical University, Guangzhou, China

**Keywords:** CRM1, NT-PGC-1α, myocardial infarction, antihypertrophy, nuclear distribution, cardiac function

## Abstract

Chromosomal maintenance 1 (CRM1) inhibitors display antihypertrophic effects and control protein trafficking between the nucleus and the cytoplasm. PGC-1α (peroxisome proliferator-activated receptor gamma coactivator-1alpha) is a type of transcriptional coactivator that predominantly resides in the nucleus and is downregulated during heart failure. NT-PGC-1α is an alternative splicing variant of PGC-1α that is primarily distributed in the cytoplasm. We hypothesized that the use of a CRM1 inhibitor could shuttle NT-PGC-1α into the nucleus and activate PGC-1α target genes to potentially improve cardiac function in a mouse model of myocardial infarction (MI). We showed that PGC-1α and NT-PGC-1α were decreased in MI-induced heart failure mice. Phenylephrine and angiotensin II were applied to induce hypertrophy in neonatal rat ventricular myocytes (NRVMs). The antihypertrophic effects of the CRM1-inhibitor Selinexor was verified through profiling the expression of β-MHC and through visualizing the cell cross-sectional area. NRVMs were transfected with adenovirus-NT-PGC-1α or adenovirus-NLS (nucleus localization sequence)-NT-PGC-1α and then exposed to Selinexor. Confocal microscopy was then used to observe the shuttling of NT-PGC-1α. After NT-PGC-1α was shuttled into the nucleus, there was increased expression of its related genes, including PPAR-α, Tfam, ERR-γ, CPT1b, PDK4, and Nrf2. The effects of Selinexor on post-MI C57BL/6j mice were determined by echocardiography and qPCR. We found that Selinexor showed antihypertrophic effects but did not influence the ejection fraction of MI-mice. Interestingly, the antihypertrophic effects of Selinexor might be independent of NT-PGC-1α transportation.

## Introduction

Rates of heart failure (HF) are rising at an alarming rate throughout the world (Martinez-Gonzalez and Ruiz-Canela, 2015). HF is commonly accompanied by cardiac hypertrophy (Warren et al., 2017). Previous studies suggest that chromosomal maintenance 1 (CRM1) (also called exportin-1) inhibitors exhibit antihypertrophic effects by influencing β-MHC and HDACs (Monovich et al., 2009; Chahine et al., 2015). Additional studies on NT-PGC-1α, which is an alternative splicing variant of PGC-1α, have revealed that CRM1 inhibitors can lead to a relative increase in the nuclear distribution of NT-PGC-1α (Chang et al., 2010). Thus, the regulation of NT-PGC-1α by CRM1 inhibitors might represent a novel mechanism of their anti-cardiac-hypertrophy effect.

NT-PGC-1α is a powerful regulator of fatty acid oxidation (FAO) in adipose tissue (Zhang et al., 2009; Jun et al., 2014; Kim et al., 2016; Liu et al., 2018). In recent years, a large amount of experimental evidence has demonstrated that HF is associated with metabolic dysfunction, which is accompanied by the down regulation of PGC-1α, a key factor in controlling mitochondrial energy metabolism (Arany et al., 2006; Schilling and Kelly, 2011; Arumugam et al., 2016). It is generally accepted that the dysregulation of mitochondrial energy metabolism aggravates HF (Chang et al., 2016; Parihar and Parihar, 2017). Thus, NT-PGC-1α might be important in the progression and pathogenesis of HF. NT-PGC-1α is predominantly distributed in the cytoplasm, and the PGC1 families fulfill most of their function through the coactivation of transcription factors in the nucleus (Zhang et al., 2009).

Although the subcellular distributions of NT-PGC-1α and PGC-1α are different, NT-PGC-1α is able to partially compensate for the function of PGC-1α, and this capability seems to be even more robust in FAO (Jun et al., 2014). Furthermore, cAMP (cyclic adenosine monophosphate) analogs can trigger both the shuffling of NT-PGC-1α into the nucleus and the activation of PGC-1α (Zhang et al., 2009). Therefore, it is plausible that NT-PGC-1α fulfills the role of PGC-1α by entering the nucleus when energy demands are increased. Importantly, the target genes of the PGC1 families are not limited to genes involved in FAO but include others, such as FOXO, ERRs, and NRFs, which are favorable in cardiovascular diseases (Vega et al., 2000; Riehle and Abel, 2012; Martin et al., 2014). Thus, we hypothesized that transporting NT-PGC-1α into the nucleus would likely lead to the activation of targets of PGC-1α, which might be beneficial to cardiac function. As a result, CRM1 inhibitors might be promising candidates for this strategy.

## Materials and Methods

### Experimental Animals

The 8- to 10-week-old male C57BL/6J mice and neonatal Sprague-Dawley rats that were used in this study were obtained from the laboratory of animal center of Southern Medical University. All mice were housed in cages and bred in a temperature-controlled room that was maintained at 22–26°C on a 12 h light–dark cycle with standard feed and water. This study was approved by the Southern Medical University Review Board and the animals used in this study comply with the Guide for the Care and Use of Laboratory Animals (NIH, 8th Edition, 2011).

### Models of Myocardial Infarction

The mice used in this study were anesthetized with a mixture of xylazine (5 mg/kg) and ketamine (100 mg/kg) delivered by intraperitoneal injection. Once the mouse was anesthetized, the trachea was intubated to provide mechanical ventilation (inspiration/expiration ratio: 1:3, 120 strokes/min), the left thorax of the mouse was opened, and a left coronary artery ligation was performed to induce a myocardial infarction. Successful ligation was confirmed by ST-segment elevation as measured by an electrocardiogram. The sham-operated mice were subjected to the same treatment without ligation. Three days after the operation, Selinexor (10 mg/kg) or DMSO (20 μl) mixed with 0.2 ml distilled water were given by gavage to MI-mice every 3 days. After 30 postoperative days, the mice were sacrificed using an overdose of anesthetic. Their hearts were extracted and soaked in liquid nitrogen, then stored in an −80°C freezer for future use or extracted for HE and immunohistochemical staining.

### Echocardiography

Echocardiography was performed on anesthetized (2% isoflurane) mice using a VEVO2100 system (Visual Sonic, North American), which has been previously described (Liu et al., 2017). The LV end-diastolic diameter (LVEDD) and LV end-systolic diameter (LVESD) were measured using the M-mode.

### Isolation and Culture of Neonatal Rat Ventricular Cardiomyocytes (NRVMs)

One- to three-day-old Sprague-Dawley rats were sacrificed via 2% isoflurane inhalation and cervical dislocation. The hearts were then removed, dissected, and enzymatically digested with 0.2% pancreatin overnight. The cells were then isolated by magnetic stirring with collagenase II (1 mg/mL) in a sterile glass vial. After 90 min of differential adhesion, the isolated cells were plated in a culture dish containing 0.1 mM 5-bromo-2′-deoxyuridine (BrdU, Sigma) and 10% fetal bovine serum (FBS, Gibco) to inhibit fibroblast proliferation. After 48 h, spontaneously contracting neonatal rat ventricular myocytes (NRVMs) were treated with either 10 μM phenylephrine (PE, Selleck) for 72 h, 1 μM angiotensin II (AngII, Abcam) for 24 h or 50 nM Selinexor (Selleck) for 4 h in Dulbecco’s Modified Eagle’s Medium (DMEM) (Gibco; Thermo Fisher) containing penicillin and streptomycin (100:1) (Gibco; Thermo Fisher). Phalloidin staining was performed by first fixing the cells with 4% paraformaldehyde for 10 min, then washing with phosphate buffered saline containing 0.1% Triton X-100, and finally staining with FITC-phalloidin (Actin-tracker green) (Beyotime, China) for 60 min and Hoechst 33258 (Thermo Fisher) for 15 min. Adenovirus-infected cells were stained Hoechst 33258 (Thermo Fisher) for 15 min after being treated with 50 nM Selinexor (Selleck) for 4 h. The results were visualized by confocal microscopy. Cell viability was assessed using the Cell Counting Kit-8 (CCK8) according to the manufacturer’s guidelines and previous reports (Lin et al., 2016). The CCK8 assay was also used to test the toxicity of Selinexor on the NRVMs.

### Infection of Adenovirus in NRVMs

The adenoviruses, including mCherry-NT-PGC-1α and mCherry-NLS-NT-PGC-1α, were purchased from the OBiO Technology Corp. Ltd., China. Nuclear localization sequence (NLS) is an amino acid sequence that controls protein import into the cell nucleus. A multiplicity of infection (MOI) of 100 was used for the adenovirus to induce the overexpression of NT-PGC-1α in the NRVMs. Four hours after the initial infection, an equal volume of fresh growth medium was added to the culture. The cells were then incubated for 24 h to allow the virus to achieve its maximum effect.

### Real-Time PCR Assays

Total RNA was extracted by RNAiso Plus (Takara). We used 1 μg of total RNA per reaction for the first-strand cDNA synthesis using RT primers (Takara). Quantitative real-time PCR assays were performed using cDNA in a 10 μL reaction volume (SYBR Green PCR kit; Takara) on an Applied LightCycler 480 system (Roche). Gene expression levels were measured using the ΔΔCt method with normalization to β-actin. The primers used in this study were obtained from Sangon (primer sequences were listed in Tables 1, 2).

**TABLE 1:** Primer sequences.

**Target genes**	**Forward primer**	**Reverse primer**
β-Actin *(Rat)*	*TGGACAGTGAGGCAAGGATAG*	*TACTGCCCTGGCTCCTAGCA*
TFAM*(Rat)*	*GCTGATGGGCTTAGAGAAGG*	*CCGAGGTCTTTTTGGTTTTC*
Acadm*(Rat)*	*GTCGCCCCAGACTACGATAA*	*GCCAAGACCACCACAACTCT*
Acadvl*(Rat)*	*TGGACAAAGGAAAGGAACTCA*	*ACTCAGACCACTGCCAATCC*
PDK4*(Rat)*	*ACCGTCGTCTTGGGAAAAG*	*CGTTGGAGCAGTGGAGTATG*
Glut4*(Rat)*	*AGGCACCCTCACTACCCTTT*	*AGCATAGCCCTTTTCCTTCC*
ERRγ*(Rat)*	*ATCCCCAGACCAAGTGTGAA*	*TGAGGCAACCCCATAGTGA*
PPARγ*(Rat)*	*CGTCCCCGCCTTATTATTCT*	*CCTGATGCTTTATCCCCACA*
NRF2*(Rat)*	*CAAATCCCACCTTGAACACA*	*TGACTAATGGCAGCAGAGGA*
Cpt2*(Rat)*	*CTGTCCACCAGCACTCTGAA*	*GCAGCCTATCCAGTCATCGT*
Cpt1b*(Rat)*	*AAGAACACGAGCCAACAAGC*	*TACCATACCCAGTGCCATCA*
PPAR-α*(Rat)*	*GACAAGGCCTCAGGATACCA*	*TCTTGCAGCTTCGATCACAC*
		

**TABLE 2:** Primer sequences.

**Target genes**	**Forward primer**	**Reverse primer**
Nrf-1*(mouse)*	*GCCAATGTCCGCAGTGAT*	*ACGGTCTGTGATGGTACGAG*
Nrf-2*(mouse)*	*GGCCACTTAAAAGACGAGA*	*GACTTCAAGATACAAGGTGCT*
ERR-α*(mouse)*	*CGGTGTGGCATCCTGTGA*	*GCGTCTCCGCTTGGTGAT*
ERR-γ*(mouse)*	*TCTTGACAGAGTGCGTGGAG*	*CACCAACAAATGCGAGACAA*
TFAM*(mouse)*	*GCTGATGGGTATGGAGAAGG*	*GCTGAACGAGGTCTTTTTGG*
PGC-1α*(mouse)*	*ACGCAGCCCTATTCATTGTT*	*TCCTTTGGGGTCTTTGAGAA*
CPT1b*(mouse)*	*GCACACCAGGCAGTAGCTTT*	*CAGGAGTTGATTCCAGACAG GTA*
CPT2*(mouse)*	*CAGCACAGCATCGTACCCA*	*TCCCAATGCCGTTCTCAAAAT*
Acadvl*(mouse)*	*GTTCCCATACCCATCTGTGC*	*GTGTCGTCCTCCACCTTCTC*
Acadm*(mouse)*	*TTGAGTTGACGGAACAGCAG*	*TTGATGAGAGGGAACGGGTA*
SOD2*(mouse)*	*TCTCAACGCCACCGAGG*	*AGACCCAAAGTCACGCT*
SOD3*(mouse)*	*TAGGACGACGAAGGGAGGT*	*GGTCCCCGAACTCATGC*

### Western Blot and Co-immunoprecipitation

Proteins were collected from the cultured NRVMs and murine hearts by a mixture of RIPA buffer (Beyotime Biotechnology, Shanghai, China) with protease inhibitor (Sigma, United States) (1:100) and quantified by BCA assay (Thermo Fisher). The primary antibodies used included Anti-β-MHC (1:1000, Abcam, United States), Anti-N-terminal NT-PGC-1α (1:1000, Abcam, United States), anti-CRM1 (1:500, Abcam, United States) and anti-β-actin (1:1000, Bioss, Beijing, China). The secondary antibody used was a goat anti-rabbit IgG-HRP (1:5000, Santa Cruz, United States). Immunoreactive bands were detected by the Pierce ECL Substrate (Pierce Biotechnology, Rockford, IL, United States) and Gene Gnome Imaging System (Syngene Bio Imaging) and quantified with the NIH ImageJ software package. The protein A/G agarose beads were purchased from Santa Cruz Biotechnology, and the CO-IP protocol was carried out according to the manufactures’ guidelines.

### Statistical Analyses

Quantitative data were displayed as the mean ± SEM (standard error of mean). A normal distribution test was performed to determine if parametric or non-parametric tests should be used. Comparisons between the two experimental groups were based on a two-tailed *t*-test while comparisons of the parameters across more than three groups were analyzed by ANOVA followed by a Dunnett’s T3 for *post hoc* multiple comparisons. For all analyses, differences were considered to be statistically significant at a value of *P* < 0.05.

## Results

### Downregulation of PGC-1α and NT-PGC-1α in Mice With MI-Induced Heart Failure

**FIGURE 1:**
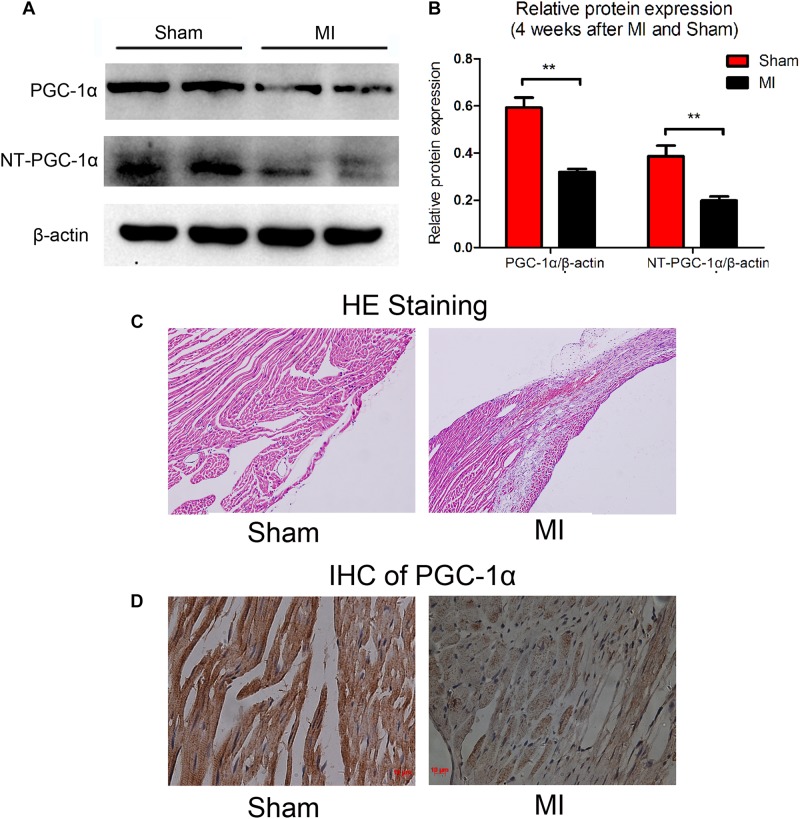
Decreased levels of PGC-1α and NT-PGC-1α in myocardial infarction mice. A representative western blot **(A)** and relative quantification to β-actin **(B)** of PGC-1α and NT-PGC-1α in mice subjected to a sham operation or MI. ^∗∗^*p* < 0.01 compared to the sham group, *n* = 4–5 in each group (*t*-test). The representative photomicrographs of HE staining **(C)** and IHC of total PGC-1α **(D)** in sham and MI mice.

Previous studies have thoroughly described alterations in cardiac metabolic substrates during HF. Here, we used a model of HF that was induced by MI. Four weeks after the operation, the myocardial expression of PGC-1α and NT-PGC-1α were significantly decreased (compared to sham-operated mice; *n* = 5; *P* < 0.05), as determined by Western blot (Figures 1A,B). The representative photographs of the immunohistochemical staining are shown in Figures 1C,D.

### Antihypertrophic Effects of the CRM1-Inhibitor Selinexor

**FIGURE 2:**
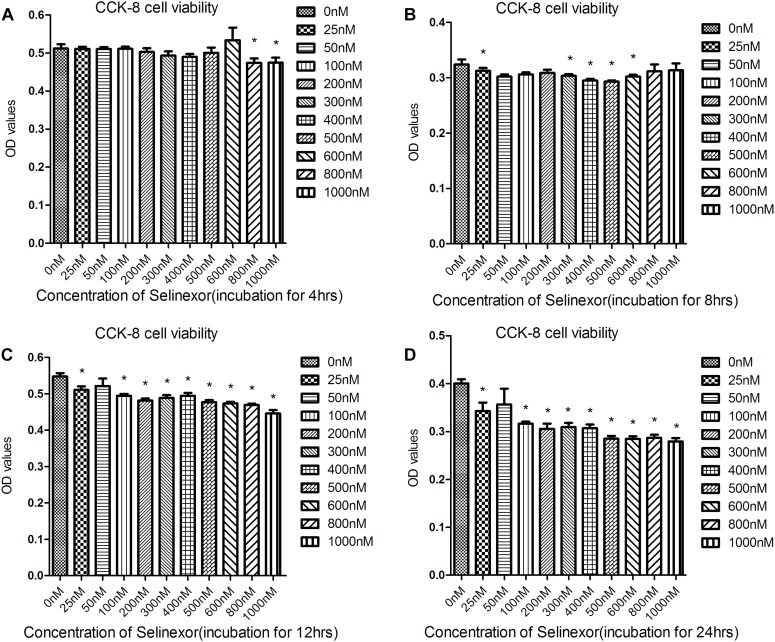
Results of the Cell Counting Kit-8 assay to determine cell viability after exposure to Selinexor. NRVMs were exposed to different concentrations of Selinexor and incubated for **(A)** 4 h, **(B)** 8 h, **(C)** 12 h, and **(D)** 24 h, respectively. *P < 0.05, compared to the corresponding control group.

**FIGURE 3:**
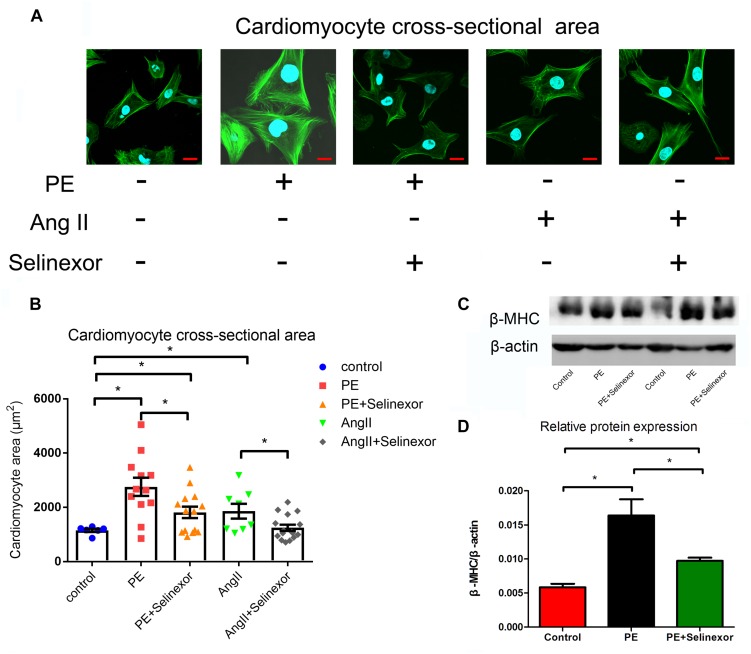
Effects of the CRM1-inhibitor Selinexor on cardiac hypertrophy. **(A)** Representative photomicrographs of the actin-tracker green stain in NRVMs that are exposed to PE, AngII and Selinexor, and **(B)** their relative cross-sectional areas. **(C,D)** The expression of β-MHC in cells that were stimulated by PE and Selinexor as detected by western blot. *P < 0.05, compared to the corresponding control group (*n* = 4).

We aimed to determine an appropriate concentration of the CRM1-inhibitor Selinexor for NRVMs. Cells were exposed to different concentrations of Selinexor for a range of durations and NRVM viability was assayed using the CCK8 cell viability assay. We discovered that cells exposed to less than 600 nM Selinexor for 4 h did not show any significant differences compared to controls (Figures 2A–D). However, a prolonged reaction time with Selinexor led to decreased OD values. Thus, we applied 50 nM Selinexor to stimulate the NRVMs for 4 h. The antihypertrophic effect of CRM1 inhibitors was demonstrated in previous studies (Harrison et al., 2004; Monovich et al., 2009; Chahine et al., 2015). Here, we verified that Selinexor can restrict PE- and AngII-induced hypertrophy in NRVMs and visualized the cell’s cross-sectional areas using Phalloidin staining and confocal microscopy (Figure 3A). The area of the NRVMs is displayed using a micrometer scale (μm^2^). As expected, the control group was smaller compared to the PE group (1,148.89 ± 73.85 μm^2^ vs. 2,756.683 ± 333.48 μm^2^, *p* < 0.01) and the AngII group (1,148.89 ± 73.85 μm^2^ vs. 1,861.60 ± 243.38 μm^2^, *p* < 0.05), while the PE group had a larger cell cross-sectional area than the PE+Selinexor group (2,756.683 ± 333.48 μm^2^ vs. 1,818.56 ± 209.08 μm^2^, *p* < 0.05). Similarly, the AngII group had a larger cell cross area compared to the AngII + Selinexor group (1,861.60 ± 243.38 vs. 1,247.71 ± 113.65, *p* < 0.05) (Figure 3B). Further investigation showed that Selinexor can inhibit the expression of β-MHC that is induced by PE (PE vs. PE+Selinexor: 0.01637 ± 0.00239 vs. 0.00973 ± 0.00047, *p* < 0.05) (Figures 3C,D). These results show that the CRM1-inhibitor Selinexor, which displays oral activity, can restrict cardiac hypertrophy *in vitro*.

### Regulation of NT-PGC-1α Distribution by CRM1 Inhibitor and NLS (Nucleus Localization Sequence)

**FIGURE 4:**
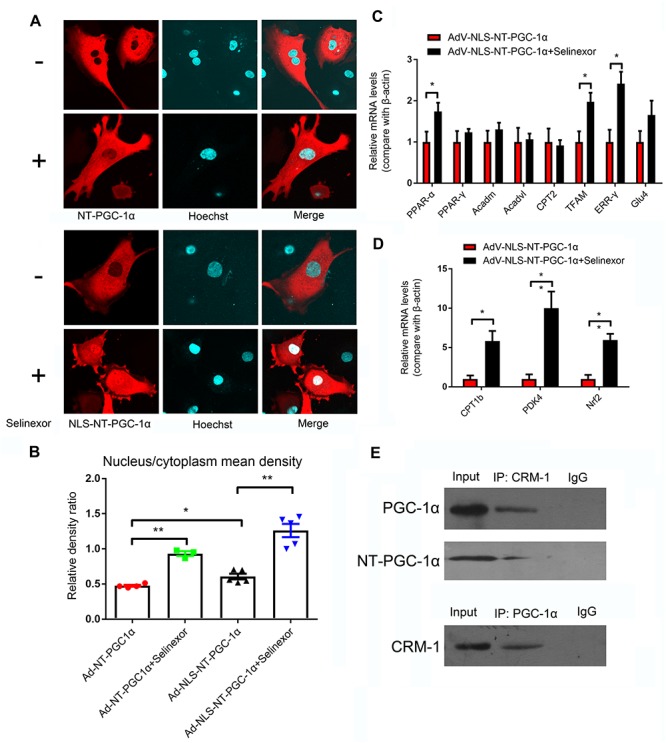
Regulation of the distribution of NT-PGC-1α by the CRM1 inhibitor and relative mRNA expression after NT-PGC-1α shuttling into the nucleus. **(A)** The influence of the CRM1 inhibitor Selinexor and the NLS (nuclear localization sequence) on the subcellular distribution of NT-PGC-1α, and **(B)** the mean density ratio of the nucleus/cytoplasm. *P < 0.05, **P < 0.05 compared to the control group, *n* = 3–5 in each group. **(C,D)** The relative mRNA expression after NT-PGC-1α is shuttled into the nucleus by Selinexor and NLS in NRVMs that are overexpressing NLS-NT-PGC-1α. *P < 0.05, **P < 0.01 compared to the corresponding group with NLS-NT-PGC-1α overexpression, *n* = 6–9 in each group. **(E)** Co-IP of PGC-1α, NT-PGC-1α, and CRM1.

Neonatal rat ventricular myocytes were transfected with adenovirus-mCherry-NT-PGC-1α and adenovirus-mCherry-NLS-NT-PGC-1α to investigate the role of CRM1 inhibitors in the regulation of NT-PGC-1α. Following the infection, the cells were then treated with 50 nM Selinexor. After stimulation, the cells were stained with Hoechst 33258 and visualized with confocal microscopy. We determined that Selinexor and NLS can increase the nuclear density of mCherry, and the nucleus/cytoplasm mean densities were also measured. Comparisons between the AdV-NT-PGC-1α and AdV-NT-PGC-1α+Selinexor groups showed significant differences (0.48 ± 0.01 vs. 0.93 ± 0.03, respectively, *p* < 0.001); the AdV-NLS-NT-PGC-1α group had lower mean density than the AdV-NLS-NT-PGC-1α+Selinexor group (1.26 ± 0.09 vs. 0.61 ± 0.04, respectively, *p* < 0.001), while comparisons between the AdV-NT-PGC-1α and Adv-NLS-NT-PGC-1α group showed significant differences (0.48 ± 0.01 vs. 0.61 ± 0.04, respectively, *p* < 0.05) (Figures 4A,B). Furthermore, we discovered that CRM1 interacted with NT-PGC-1α and PGC-1α in the NRVMs through the use of Co-IP (Figure 4E). These findings suggest that the trafficking of NT-PGC-1α between the nucleus and cytoplasm are likely dependent on the interaction between CRM1 and its corresponding protein.

### Relative mRNA Expression After Shuttling NT-PGC-1α Into the Nucleus

We discovered that Selinexor and NLS could dramatically increase the nuclear distribution of NT-PGC-1α. Thus, we explored relative mRNA expression after the shuttling of NT-PGC-1α into the nucleus. We discovered that the NRVMs that were transfected with AdV-NLS-NT-PGC-1α and exposed to Selinexor could increase their relative amount of mRNA expression compared to cells that were transfected with AdV-NLS-NT-PGC-1α (Figures 4C,D). These genes include PPAR-α (1.00 ± 0.25 vs. 1.74 ± 0.21, *p* < 0.05); Tfam (1.00 ± 0.26 vs. 1.98 ± 0.22, *p* < 0.05); ERR-γ (1.00 ± 0.30 vs. 2.42 ± 0.29, *p* < 0.01); CPT1b (1.00 ± 0.01 vs. 5.82 ± 1.28, *p* < 0.05); PDK4 (1.00 ± 0.58 vs. 10.00 ± 2.12, *p* < 0.05); and Nrf2 (1.00 ± 0.52 vs. 5.96 ± 0.78, *p* < 0.001). These results suggest that NT-PGC-1α might function in nuclear transcription, and could partially compensate for the function of full-length PGC-1α. Additionally, most of the genes mentioned above have beneficial cardiovascular effects as reported by previous studies.

### The Influence of Selinexor on Cardiac Function and mRNA Expression in MI-Induced Heart Failure Mice

**FIGURE 5:**
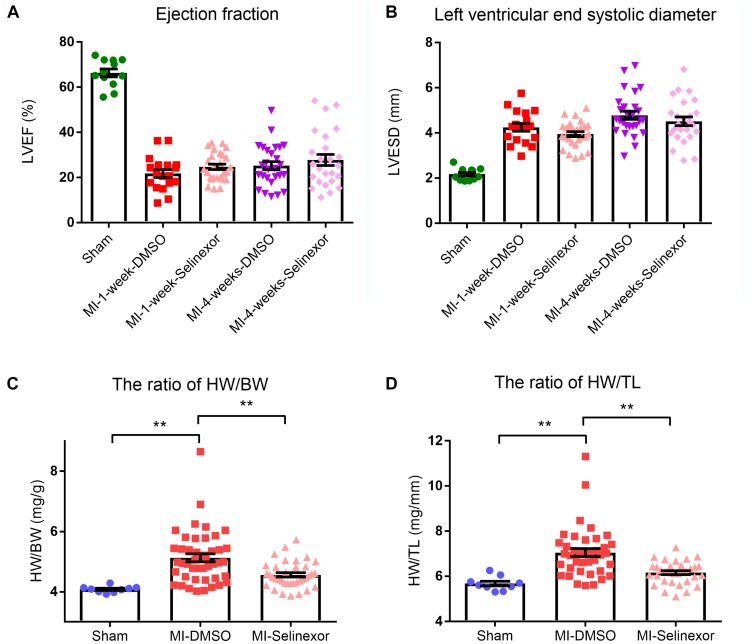
Echocardiography results and HW/BW and HW/TL ratios in MI-mice treated with Selinexor. **(A)** The ejection fraction **(B)** in MI-mice treated with Selinexor or vehicle post-myocardial infraction was detected by echocardiogram at 1 week and at 4 weeks (*n* = 12, 18, 27, 27, and 25 in sham mice, MI-1-week-DMSO, MI-1-week-Selinexor, MI-4-week-DMSO, MI-4-week-Selinexor, respectively). The ratio of **(C)** HW/BW and **(D)** HW/TL in MI-mice gavaged with Selinexor (**P < 0.01 compared to the corresponding group).

**FIGURE 6:**
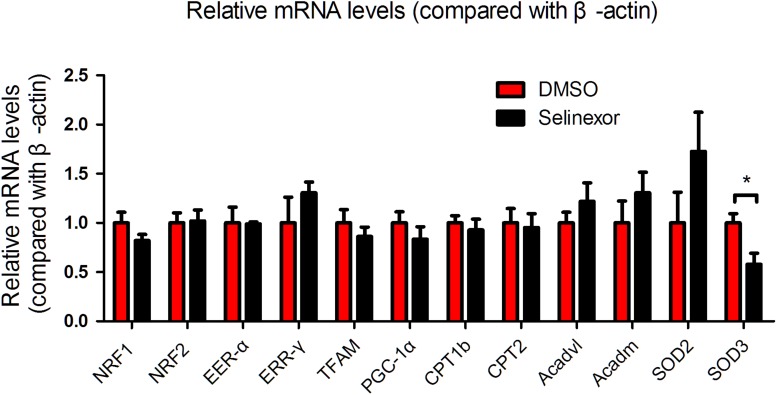
mRNA expression in MI-mice treated with Selinexor. Cardiac mRNA expression in WT mice treated with Selinexor and vehicle post-MI at 1 month (*n* = 5 in each group, *P < 0.05 compared to the corresponding group).

If CRM1 inhibitors exhibit cardiovascular protective effects, they might be due to their antihypertrophic effects and the shuttling of NT-PGC-1α into the nucleus. To test this hypothesis, we gavaged Selinexor to C57BL/6j mice that then underwent coronary artery ligation. If Selinexor could ameliorate energy metabolism *in vivo*, then it would likely enhance the ejection fraction (EF) or reduce the left ventricular end-systolic diameter (LVESD) in mice. We performed an echocardiogram on these mice at 1 week and 4 weeks post-myocardial infraction but the results were not significant between the Selinexor and control groups (Figures 5A,B and Supplementary Table S1). However, we found that the Selinexor treatment reduced HW/BW (the ratio of heart weight and body weight) and HW/TL (the ratio of heart weight and tibia length) in the MI-mice (Figures 5C,D), which is in line with the cellular findings. Furthermore, there were no significant differences in the relative mRNA levels of PGC-1α between these groups, except for SOD3 (Figure 6). These findings indicate the potential effects of Selinexor in heart remodeling.

## Discussion

In the present study, we suggest that the antihypertrophic effects of the CRM1 inhibitor Selinexor might be independent of NT-PGC-1α. We found that PGC-1α and NT-PGC-1α were decreased in the MI-induced HF mouse model, which supports the role of energy metabolism defects in HF (Warren et al., 2017). Although the mechanisms of PGC-1α have been fully elucidated, there are relatively few studies that have investigated the role of NT-PGC-1α in the heart (Liu et al., 2018). Additionally, the trafficking of NT-PGC-1α between the nucleus and the cytoplasm by CRM1 inhibitors in previous studies was only demonstrated in other cell lines (Chang et al., 2010). Therefore, CRM1 might be a promising target in HF due to its antihypertrophic effects and its regulation of NT-PGC-1α.

We detected the antihypertrophic effects of a CRM1 inhibitor that have been confirmed in previous studies (Harrison et al., 2004; Monovich et al., 2009; Chahine et al., 2015). Our results suggest that the CRM1 inhibitor Selinexor can alleviate PE- and AngII-induced cardiac-hypertrophy and inhibit the expression of β-MHC induced by PE. Thus, it is plausible that the antihypertrophic effects of Selinexor are achieved by trafficking NT-PGC-1α between the nucleus and the cytoplasm.

To explore the role of nuclear NT-PGC-1α, we utilized an adenovirus that expresses a NLS-fused protein to infect NRVMs and treated the NRVMs with Selinexor. We found that cells exposed to Selinexor slightly increase their nuclear concentration of NT-PGC-1α, but cells that express NLS-NT-PGC-1α and are exposed to Selinexor dramatically increase their nuclear content of NT-PGC-1α. These results agree with previous studies that showed CRM1 inhibitors can lead to the retention of nuclear NT-PGC-1α levels (Chang et al., 2010; Shen et al., 2012). Zhang et al. (2009) suggested that NT-PGC-1α lacked the structure of the NLS in its C terminus and that, under the influence of the nuclear exporting sequence (NES), NT-PGC-1α is predominantly distributed in cytoplasm. The CRM1 protein affects protein trafficking between the nucleus and the cytoplasm (Lu et al., 2015), which is a reported mechanism in the regulation of NT-PGC-1α subcellular distribution (Chang et al., 2010; Shen et al., 2012). Chang et al. (2010) reported that the CRM1-inhibitor leptomycin B (LMB) resulted in NT-PGC-1α being retained in the nucleus and increase its nuclear concentration.

In this study, we confirmed the interaction between CRM1 and both NT-PGC-1α and PGC-1α by immunoprecipitation. Furthermore, we showed that the expression of mRNAs involved in mitochondrial function are increased after NT-PGC-1α is shuttled into the nucleus. Collectively, these data indicate a possible mechanism of a CRM1 inhibitor in the heart, by which the retention of NT-PGC-1α in the nucleus is able to respond to cellular energy demands.

However, *in vivo* applications of Selinexor did not improve LV ejection fractions, had no effect on the expression of genes involved in energy metabolism, and even reduced the expression of SOD3, which indicates that it might be involved with other mechanisms and could be associated with toxicity. Nevertheless, Selinexor reduced the ratios of HW/BW and HW/TL, which likely fulfills an antihypertrophic role that is independent of NT-PGC-1α shuttling.

There are some plausible explanations for the *in vivo* results. First, the CRM1 inhibitor is unable to dramatically increase the levels of nuclear NT-PGC-1α without a fused NLS, and mice do not have the fused NLS in NT-PGC-1α. Second, myocardial infraction is accompanied by severe oxidative stress, which might dampen the expression of NT-PGC-1α. This is supported by previous reports of the inhibition of NT-PGC-1α by H_2_O_2_-treated medium (Choi et al., 2014). Third, high doses of Selinexor might induce toxicity, which would restrict higher doses in mice. Although we failed to identify any significant effects of the CRM1 inhibitor on cardiac energy metabolism, the shuttling of NT-PGC-1α into the nucleus remains a possible method to ameliorate metabolic dysfunction (Chang and Ha, 2017). Additionally, recent studies show that cytoplasmic NT-PGC-1α interacts with some mitochondrial proteins (Choi et al., 2013; Chang and Ha, 2017), which indicates its bilateral effects in both the nucleus and the cytoplasm. Further studies are required to determine if the retention of NT-PGC-1α in the nucleus is associated with any additional impairments.

## Conclusion

Taken together, our findings indicate that Selinexor enhances the expression of several metabolic genes in the presence of NT-PGC-1α overexpression when using an *in vitro* model and that its antihypertrophic effects might be independent of NT-PGC-1α shuttling.

## Ethics Statement

This study was carried out in accordance with the recommendations of the guidelines of the Animal Care Committee of Nanfang Hospital, Southern Medical University. The protocol was approved by the Animal Care Committee of Nanfang Hospital.

## Author Contributions

ZL, HT, JH, WC, YB, QcZ, QoZ, WL, HR, and DX made a substantial contribution to the conception or design of the work or the acquisition, analysis, or interpretation of data for the work; approved the final version to be published; and also agreed to be accountable for all aspects of the work in ensuring that questions related to the accuracy or integrity of any part of the work are appropriately investigated and resolved. ZL, HT, HR, and DX drafted the work or revised it critically for important intellectual content.

## Conflict of Interest Statement

The authors declare that the research was conducted in the absence of any commercial or financial relationships that could be construed as a potential conflict of interest.

## Supplementary Material

TABLE S1 The echocardiography of MI-mice.
